# Neuroimaging in Fabry disease: current knowledge and future directions

**DOI:** 10.1007/s13244-018-0664-8

**Published:** 2018-11-02

**Authors:** Sirio Cocozza, Camilla Russo, Giuseppe Pontillo, Antonio Pisani, Arturo Brunetti

**Affiliations:** 1 Department of Advanced Biomedical Sciences, University “Federico II”, Via Pansini, 5, 80131 Naples, Italy; 2 Department of Public Health, Nephrology Unit, University “Federico II”, Via Pansini 5, 80131, Naples, Italy

**Keywords:** Fabry disease, Central nervous system, Magnetic resonance imaging

## Abstract

**Abstract:**

Fabry disease (FD) is a rare X-linked disorder characterised by abnormal progressive lysosomal deposition of globotriaosylceramide in a large variety of cell types. The central nervous system (CNS) is often involved in FD, with a wide spectrum of manifestations ranging from mild symptoms to more severe courses related to acute cerebrovascular events. In this review we present the current knowledge on brain imaging for this condition, with a comprehensive and critical description of its most common neuroradiological imaging findings. Moreover, we report results from studies that investigated brain physiopathology underlying this disorder by using advanced imaging techniques, suggesting possible future directions to further explore CNS involvement in FD patients.

**Teaching Points:**

*• Conventional neuroradiological findings in FD are aspecific.*

*• White matter hyperintensities represent the more consistent brain imaging feature of FD*

*• Abnormalities of the vasculature wall of posterior circulation are also consistent features.*

*• The pulvinar sign is not reliable as a finding pathognomonic for FD.*

*• Advanced imaging techniques have increased our knowledge about brain involvement in FD.*

## Introduction

Fabry disease (FD) is a multiorgan X-linked lysosomal storage disorder caused by mutation in the *GLA* gene, which encodes for the α-galactosidase A (α-GalA) enzyme [1–4]. Its defective activity leads to intracellular accumulation of glycosphingolipids, mainly globotriaosylceramide (Gb3) [5]. Renal failure, cardiomyopathy, and central nervous system (CNS) alterations are the main causes of morbidity and reduced life expectancy in these patients, although enzyme replacement therapy (ERT) in addition to supportive treatments have had a positive impact on FD clinical management [6].

In brain tissue, Gb3 storage primary occurs in endothelium and vascular smooth muscle cells [7], but it is also responsible for glial deposition and neuronal ballooning in cortical regions and deep nuclei [8–10].

CNS symptoms can vary from very mild to severe, including manifestations related to acute cerebrovascular events and posterior circulation alterations, along with neuropathic pain, cochleo-vestibular dysfunction and a various degree of cognitive impairment and psychiatric symptoms [11].

Magnetic resonance imaging (MRI) is the reference imaging technique to evaluate possible brain damage in FD. Modern conventional MRI allows for a proper and accurate estimation of the pattern and the degree of brain alterations in patients with or without a clinical evidence of focal neurological impairment. On the other hand, different advanced MRI techniques have been applied to the study of FD patients, to better elucidate pathophysiological mechanisms underlying CNS involvement in this disorder.

Here we describe the “state of the art” in FD brain imaging, with a detailed depiction of conventional imaging findings, detailing possible pitfalls and pathognomonic relevance of the most pictorial features. Furthermore, we highlight the role of advanced techniques in providing new insights and future perspectives on CNS involvement in this condition.

## Conventional imaging

Non-invasive imaging modalities are widely used to assess type and extension of CNS damage in FD patients. While computed tomography (CT) application is only limited to acute cerebrovascular events, conventional MRI is the “gold standard” imaging technique to evaluate brain alterations in FD, being the most sensitive technique to assess type and extent of CNS involvement both in symptomatic and asymptomatic subjects. However, MRI findings can be very heterogeneous, with an overall poor global diagnostic accuracy both in terms of specificity and sensitivity. For this reason, when evaluating brain images of FD patients, it is mandatory not only to have a good knowledge of all the radiological findings present in this condition but also an accurate evaluation of both systemic organ involvement and familial history, which is known to be useful in the diagnostic workup of patients with suspected FD [12]. A summary of the major conventional imaging findings in FD is shown in Table 1.

**Table 1:** The major conventional imaging findings in Fabry disease

	Conventional imaging findings			
Stroke	Increased prevalence of cerebrovascular events in FD, with predilection for females and young subjects [11]	Frequently occurring before the diagnosis, in absence of other signs and symptoms of the underlying disorder [13]	Neurological and neuroradiological findings classical and consistent with affected vascular territory [14]	Haemorrhagic stroke is rare, whereas cerebral micro-bleeding more commonly observed (11–30% of cases) [15]
White matter hyperintensities	Observed in up to 80% of patients [16], white matter hyperintensities are the most common neuroradiological finding in FD	Non-specific distribution, with a variable periventricular, deep and/or subcortical white matter involvement [17]	High lesion load can mimic other conditions, including multiple sclerosis. Relative sparing of midline and infratentorial structures can help in the differential diagnosis [18, 19]	Long-term enzyme replacement therapy can apparently stabilise white matter lesion load [20–22]
Vertebro-basilar diameter	Dilative arteriopathy of the vertebro-basilar system is a common, although inconstant, neuroradiological feature of FD [23]	Vessel alterations include elongation, tortuosity, ectasia and focal aneurismal dilatation of vertebral and basilar arteries [24]	Basilar artery elongation and dilatation seems to be an age-dependent phenomenon, more pronounced in FD patients [25]	The increase in arterial diameters apparently show stability over time [26]
Pulvinar sign	Unilateral or bilateral hyperintensity of the thalamic pulvinar on unenhanced T1-weighted brain MRI [27]	Originally it was thought to be a common and pathognomonic sign of FD [28]	Is a sign present in a low proportion of patients (3% of cases), mostly observed in male patients with severe renal involvement [29]	Pulvinar sign should no longer be recognised as a neuroradiological finding characteristic of FD, due to its low incidence and specificity

### Stroke

Ischaemic stroke is the principal cause of premature death and permanent disability in FD [23]. Its incidence is higher than that reported in the normal population, with a slightly predilection for female patients rather than males, even in absence of a classic FD phenotype [13, 30]. It is usually interpreted as the expression of medium- and large-vessel vasculopathy, although in some cases secondary to a multifactorial involvement of other organs (e.g. atrial fibrillation, valvulopathy, etc.) [31].

Ischaemic strokes, along with silent brain infarcts and transient ischaemic attacks, are the most common manifestations in FD patients. Conversely, haemorrhagic stroke is considered a rare event in FD, being present in only around 10% of cases, with a predilection for male patients [13]. On the other hand, cerebral microbleeds are considered more common, being observed in a percentage of cases ranging from 11 to 30% of FD patients, independently from CNS involvement symptoms [15, 32]. This evidence further strengthens the hypothesis of primary endothelial dysfunction due to abnormal lipid storage in perivascular smooth muscle cells, in accordance with alterations in plasmatic endothelial biomarkers [33].

**Fig. 1:**
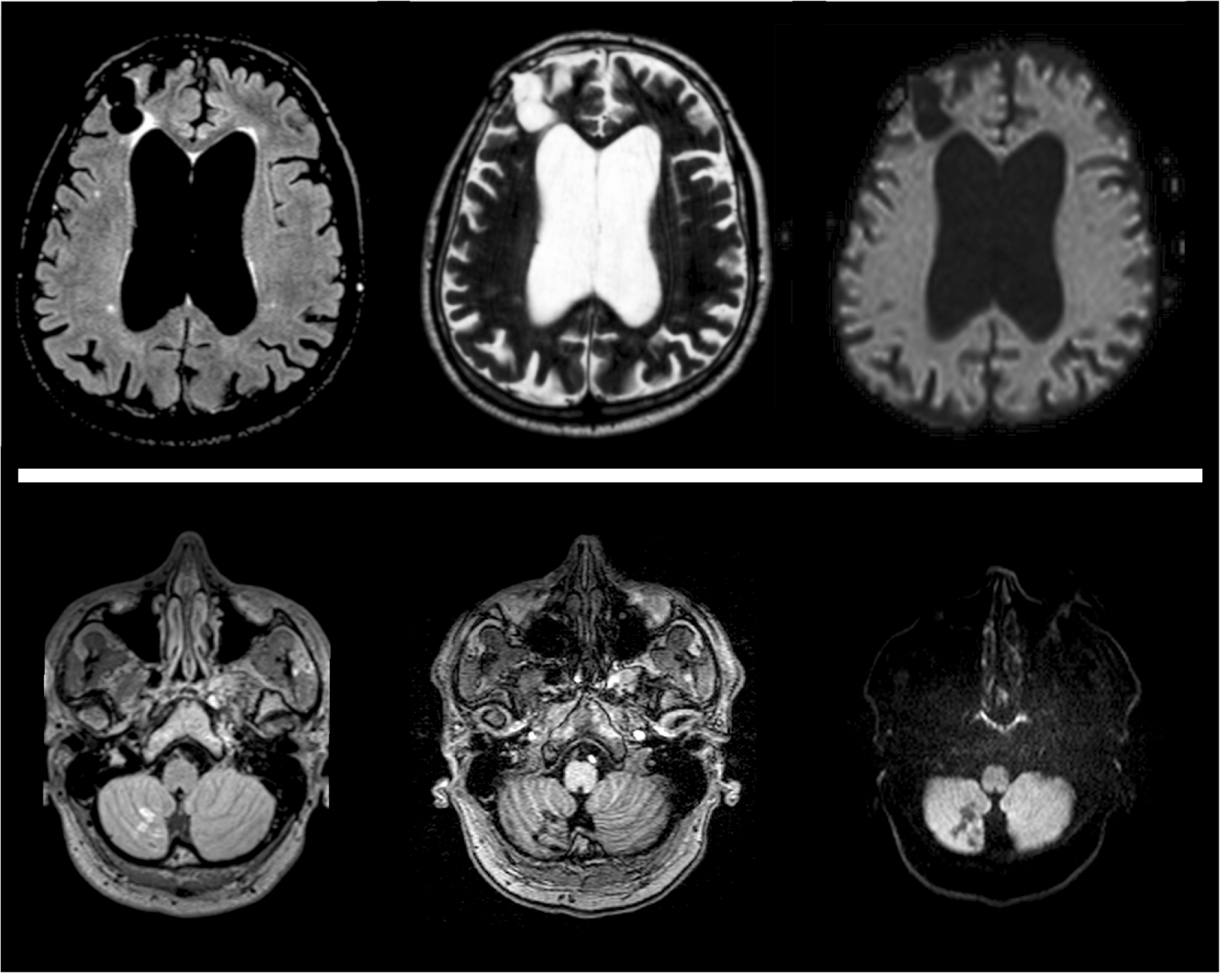
Brain MRI findings in Fabry disease patients suffering from major cerebrovascular events. In the *upper row*, images obtained from a 61-year-old woman showing the presence of a large chronic ischaemic lesion at the level of the right frontal lobe, coupled to a significant enlargement of lateral ventricles, while in the *lower row* small ischaemic lesions at the level of the inferior portion of the right cerebellar lobe are present in 52-year-old man

Imaging features of stroke in FD patients does not differ from those present in similar cases with different aetiologies, both regarding CT and MRI scans, with a comparable clinical and radiological evolution in absence of a timely intervention [14]. Conventional MRI sequences, including but not limited to diffusion-weighted imaging (SWI), T2-weighted and fluid attenuation inversion recovery (FLAIR) sequences, are generally used to detect and date ischaemic stroke (Fig. 1), while T2*-weighted and susceptibility-weighted imaging (SWI) are mainly used for characterising haemorrhagic transformation.

### White matter hyperintensities

**Fig. 2:**
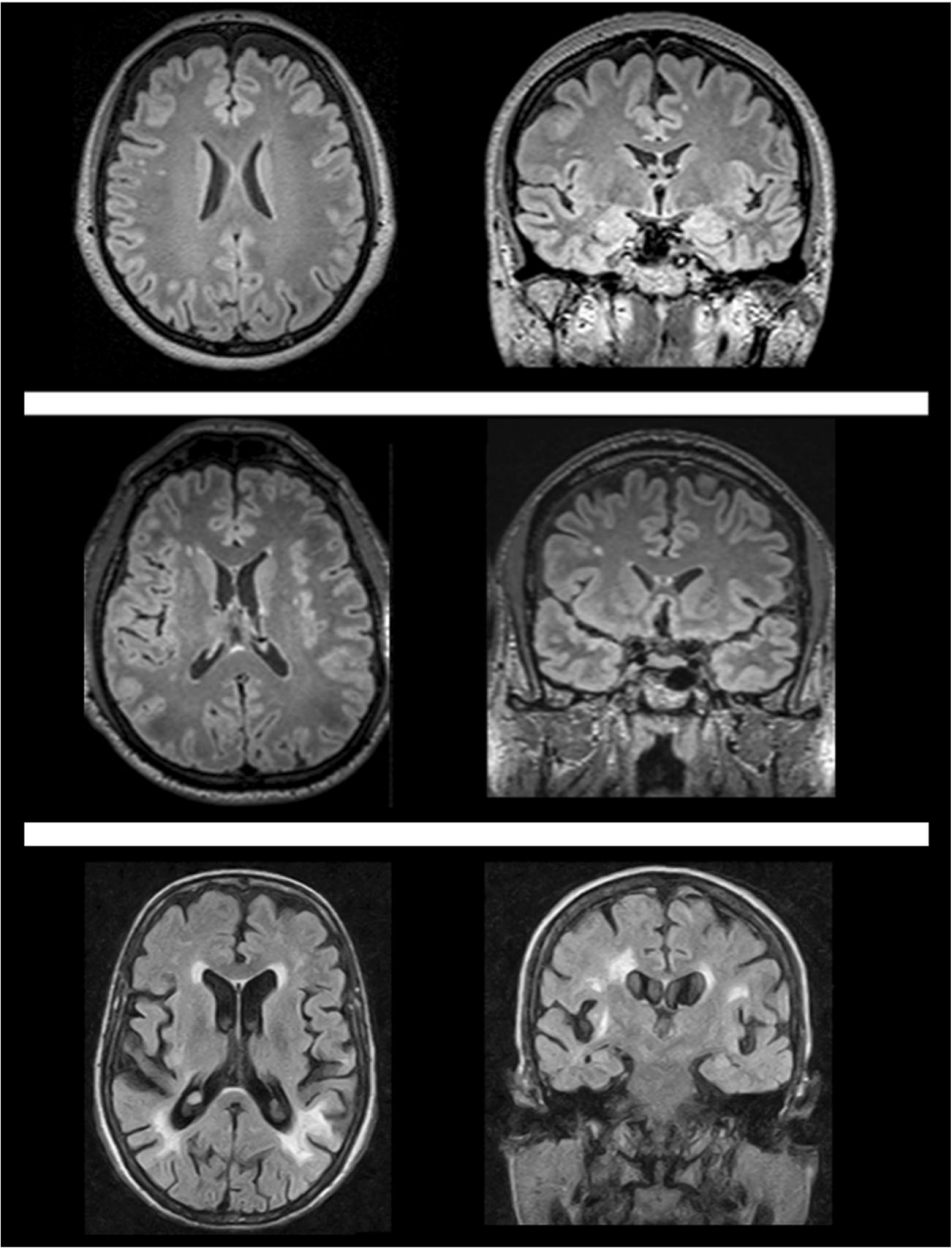
White matter involvement in Fabry disease could range from small, scattered and punctuate T2-weighted hyperintense foci (*upper row*, 46-year-old woman, or *middle row*, 52-year-old man) to bilateral diffuse, patchy and partly confluent white matter hyperintensities (*lower row*, 40-year-old woman). Although being the most common neuroimaging finding in Fabry disease, white matter hyperintensities appearance and distribution are not specific in this condition

Small vessel microangiopathy is the other most common expression of cerebral vasculopathy in FD. Indeed, up to 80% of these patients show a variable degree of periventricular, deep and/or subcortical white matter (WM) involvement [16]. The definition of WM hyperintensities (WMH) indicates lesions not referable to focal acute cerebrovascular events, and can be observed even in absence of focal neurological signs [17], representing the more consistent brain-imaging feature of FD (Fig. 2).

Mechanisms underlying the small vessel microangiopathy in FD are still unclear. It has been suggested that alterations in nitric oxide pathway, impaired arterial reactivity and subsequent occlusive microangiopathy secondary to Gb3 deposition could explain WM vulnerability [8, 34–36]. On MRI, WMH are well-recognised focal or diffuse, sometimes confluent, areas of high signal intensity on both T2-weighted and FLAIR sequences. WMH present a similar frequency in male and female patients, are generally sparse and unspecific and, although it has been described that the damage could initially occur in hypo-metabolic hyper-perfused regions [31, 37], no specific predilection for any brain region has been reported [38]. Long-term ERT can apparently stabilise WMH load, also reducing the incidence of other CNS complications [20], although further studies are required to confirm this finding [21, 22]. In some cases, a high WMH load with periventricular leukoaraiosis and diffuse leukoencephalopathy can also be observed. Therefore, a differential diagnosis with other disorders showing a similar WM involvement can be challenging [11]. In particular, FD has been described as one of the possible mimickers of multiple sclerosis (MS), as well as other demyelinating disorders, with different evidences of misdiagnosis between FD and MS reported in literature [39–46]. Considering that typical MS and FD presentations and clinical findings are very different, an appropriate anamnestic data collection along with an accurate evaluation of multi-organ damage including a proper neuroradiological evaluation is usually sufficient to lead to a correct diagnosis. However, in some cases a correct diagnosis can be more challenging, and the search for additional neuroradiological clues such as the relative sparing of midline [18] and infratentorial [19] structures may be helpful to differentiate FD from MS.

### Vertebro-basilar diameter

**Fig. 3:**
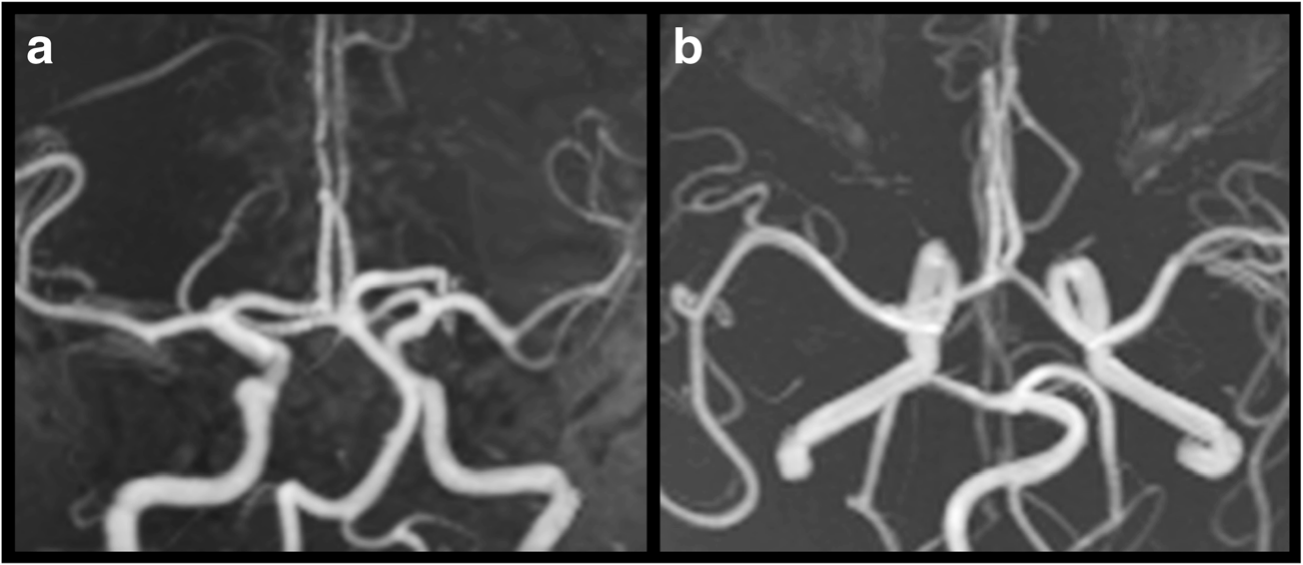
Maximum intensity projection of an intracranial three-dimensional time-of-flight MR angiography sequence acquired in a 52-year-old man with Fabry disease. Coronal (**a**) and axial (**b**) reconstructions show the presence of a mild elongation and tortuosity of the posterior circulation

The dilative arteriopathy of the vertebro-basilar system is another common, although inconstant, neuroradiological feature of FD, being documented in different previous reports [23–25, 47, 48]. Alterations of the posterior circulation system include elongation, tortuosity, diffuse ectasia and/or focal aneurismal dilatation of vertebral and basilar arteries, routinely detected with the use of a time-of-flight (TOF) MRI angiography (Fig. 3). In selected cases, integrative information can be obtained by means of post-contrast imaging techniques like enhanced MRI angiography, CT or digital subtraction angiography.

Even if the exact prevalence of this finding in the normal population is still unknown, it has been recently purposed that basilar artery elongation and dilatation could be an age-dependent phenomenon present in both healthy controls and FD subjects, but more evident in the second group [25]. However, this increase in arterial diameters apparently shows stability over time, with no significant changes after 8-year-long follow-up [26]. In line with these suggestions, when considering young patients suffering acute stroke from different causes, alterations of the vascular anatomy of the posterior circulation were probed in all patients independently for the underlying disorders. Similarly, the prevalence of dolichoectasia of the basilar artery was proved to be similar in FD patients compared to those observed in young patients with other uncommon causes of acute cerebrovascular event [47, 49]. This evidence mitigates against a possible role of the evaluation of basilar artery enlargement as a tool for the differential diagnosis between FD and non-FD related strokes [23].

Anomalies of the vasculature wall of the posterior circulation in FD are usually asymptomatic [50]. Occasionally, posterior circulation dilatation can be also responsible for compression of anterior pons and cerebello-pontine angle neuro-vascular structures, causing symptoms such as auditory and vestibular dysfunction, trigeminal neuralgia or headache [51, 52].

The mechanism underlying such abnormal vessel dilatation is still largely unknown. The most reliable hypothesis is that the primary Gb3 accumulation within vessels’ smooth muscle cells could determine an alteration in the nitric oxide pathway, leading to a progressive media layer dysfunction with subsequent elongation/ectasia [25, 31, 35], as reported in other storage disorders like the late-onset Pompe disease [53, 54].

### Pulvinar sign

Long considered a common neuroradiological finding in FD [55], the pulvinar sign is defined as a selective signal alteration of the lateral aspect of thalamic pulvinar nucleus on MRI, presenting with symmetric or asymmetric hyperintensity on unenhanced T1-weighted brain MRI. It can also be observed as a susceptibility-induced hypointense area on T2*-weighted images, which corresponds to an increased attenuation indicating calcifications on CT scans [27, 28]. Its pathogenesis is still unclear, although the hypothesis of subtle dystrophic calcifications caused by chronic hypoperfusion secondary to microvascular alterations is to date the most accepted [11, 28, 56, 57].

Originally thought to be pathognomonic of the disease, its role has been widely reconsidered in recent years. Its real incidence has been recently settled in around 3% of FD cases, with a clear predilection for males with impaired renal function [29], while a positive pulvinar sign has been only anecdotally reported in female FD patients [58]. In this minority of cases, the pulvinar sign could be interpreted as a neuroradiological epiphenomenon of a long-term accumulation of undegraded metabolites, whose systemic manifestations gradually appear during the years. In addition to the low incidence, its specificity was also reconsidered, with a wide range of different conditions in which a spontaneous thalamic T1-weighted hyperintensity is present [59, 60]. All these pieces of evidence, taken together, lead to the conclusion that the pulvinar sign should no longer be recognised as a neuroradiological finding characteristic of FD, considering its low incidence and specificity.

## Advanced imaging

Along with the conventional techniques, other evidences regarding the investigation of the pathophysiological mechanisms behind CNS impairment in FD have been collected over recent years through the use of advanced imaging techniques. A summary of the major advanced imaging findings in FD is shown in Table 2.

**Table 2:** The major advanced imaging findings in Fabry disease

Advanced imaging findings				
Brain tissue volume	In absence of a severe cerebrovascular pathology, global normalised grey and white matter volumes are preserved in FD patients	A global reduction of the intracranial volume has been observed, suggesting the presence of an abnormal neural development [61]	Voxel-based morphometry studies showed clusters of reduced grey matter density in bilateral thalami and hippocampi [61, 62]	Hippocampal atrophy increases over years, and does not correlate with lesion load [26]
Diffusion tensor imaging	A diffuse and significant microstructural white matter involvement is present in FD [63]	Elevated total brain parenchymal average diffusion constant has been observed, independent from white matter lesions [64]	Increased mean diffusivity and reduced fractional anisotropy in frontal, parietal and temporal normal-appearing white matter [65, 66]	Voxel-based DTI showed the presence of microstructural damage affecting also the thalamus [67]
Functional MRI	Significant subtle functional changes seem to occur in FD, independently from major cerebrovascular events [63, 68]	During motor task, FD patients showed increased activation of additional cortical regions [69]	Altered corticostriatal functional connectivity was observed, suggesting a subclinical involvement of motor circuits [68]	Functional changes involve not only the motor, but also cognitive functions, with alterations of the so-called default mode network [63]
Other MRI techniques	MRI spectroscopy	Magnetisation transfer	Quantitative MRI	MRI Arterial spin labelling
	Inconstant reduction in NAA/Cr ratio in different brain areas, affecting both cortical and subcortical structures [70–72]	Reduced magnetisation transfer ratio and bound-pool fraction in the normal-appearing white matter, suggesting a decrease of myelin density [73, 74]	Increased susceptibility values in the striato-nigral pathway of FD patients [75]	Increased cerebral flow in the white matter, with particular reference to the splenium of the corpus callosum [76]
Nuclear medicine	Increased relative cerebral blood flow of the posterior and periventricular regions [35]	The altered relative cerebral blood flow seems to reverse after prolonged enzyme replacement therapy [77]	No significant global glucose metabolism changes affects the brain of FD patients [37]	Hypometabolic areas only observed in regions with infarcts or haemorrhages on MRI scans [78]

### Brain tissue volume

The presence of cerebral atrophy, defined as the morphological expression of brain tissue volume (i.e. grey matter [GM] and WM) loss and visually assessed using conventional MRI sequences, has been reported in the past as a possible neuroradiological feature of FD, although with all the limitations of a qualitative assessment of brain atrophy (e.g. low accuracy, poor reproducibility) [79]. These limitations have been overcome with the use of advanced techniques, which warrant an accurate and reproducible evaluation of brain tissue volumes.

Different quantitative volumetric MRI studies explored the possibility of brain tissue volume loss in FD patients with mild-to-moderate CNS involvement, determining whether, in the absence of a severe cerebrovascular pathology, global normalised brain tissue volumes are preserved in FD patients. In this light, brain atrophy is likely to occur only in more advanced phases of CNS involvement, characterised by the presence of brain infarcts (territorial or lacunar) and/or a significant WMH load [62, 65, 73].

Possible regional differences in GM volume have also been investigated in FD patients, mostly by means of voxel-based morphometry (VBM) analysis. Different VBM studies have been conducted in FD patients in recent years, mostly showing that no differences in terms of regional GM density were present in FD patients compared to healthy controls [63, 65, 68, 80].

Nevertheless, atrophy of specific brain regions have been reported in FD patients [61, 62]. In particular, clusters of reduced GM density have been recently reported at the level of the thalami, bilaterally [61]. Along with this brain region, hippocampal atrophy has been reported in FD patients, with bilateral hippocampi volumes that were also obtained by a manual segmentation of the structures [62]. Results remained significant after correction for age, WMH load and WM/GM volume, thus possibly reflecting a direct neuronal involvement independent from vascular pathology [62]. This finding was confirmed not only using a VBM analysis [61] but also by another study in which an 11% hippocampal volume reduction over 8 years was described, not correlating with the WMH load and thus suggesting the possible independence of these two phenomena [26].

Interestingly, a recent study showed the presence of a significant reduction of the entire intracranial volume of FD patients compared to HC [61]. This reduction was coupled to a preservation of the fractional brain tissue volumes (namely, GM, WM and cerebrospinal fluid), allowing to hypothesise the presence of a harmonious reduction of the intracranial volume in FD patients, suggesting the possible presence of an abnormal neural development in this condition [61].

All this evidence, taken together, suggests that FD could be characterised by a harmonious reduction of the intracranial volume, with a relative preservation of fractional brain tissue volumes and regional GM density, although atrophy of specific regions of the brain, such as the thalamus or the hippocampus, has been reported. Future studies are warranted to further elucidate the possible presence of global and regional brain volume differences in FD, conducted with automatic measurements using the most recent software, which are known to provide more accurate estimations of intracranial volumes [81, 82].

### Diffusion tensor imaging

The assessment of cerebrovascular damage through the evaluation of WMH using conventional MRI sequences and its grading using semi-quantitative visual rating scales is known to provide a limited accuracy [83].

Conversely, diffusion tensor imaging (DTI) has proved to be a sensitive technique for detecting brain tissue alterations, allowing for an accurate and reproducible quantification of microstructural WM changes [84].

One of the first studies focusing on cerebral diffusivity in FD demonstrated an elevated total brain parenchymal average diffusion constant in FD compared to controls, also in patients without any conventional MRI-detectable WM lesion [64]. Authors attributed this result to an increase in the amount of brain interstitial water content, possibly secondary to microvascular modifications [64].

In a ROI-based DTI analysis, increased mean diffusivity values in FD patients were found at the level of the frontal, parietal and temporal normal-appearing WM [66]. This increase, presumably due to elevated water content of brain tissue, proved some degree of independency from WM lesions, and was interpreted as a biomarker of early stages of microvascular injury [66]. This finding was confirmed in a voxel-based DTI study in which diffuse changes of WM microstructure were found, particularly affecting the periventricular regions and in the posterior portion of the thalamus [67]. In line with the previous studies, these alterations were found also in patients without significant WMH load; thus suggesting that measurable elevations in interstitial water content and myelin rarefaction most likely emerge before WM lesions are detectable on conventional images, probably due to microangiopathic alterations mainly affecting the long perforating arteries [67].

**Fig. 4:**
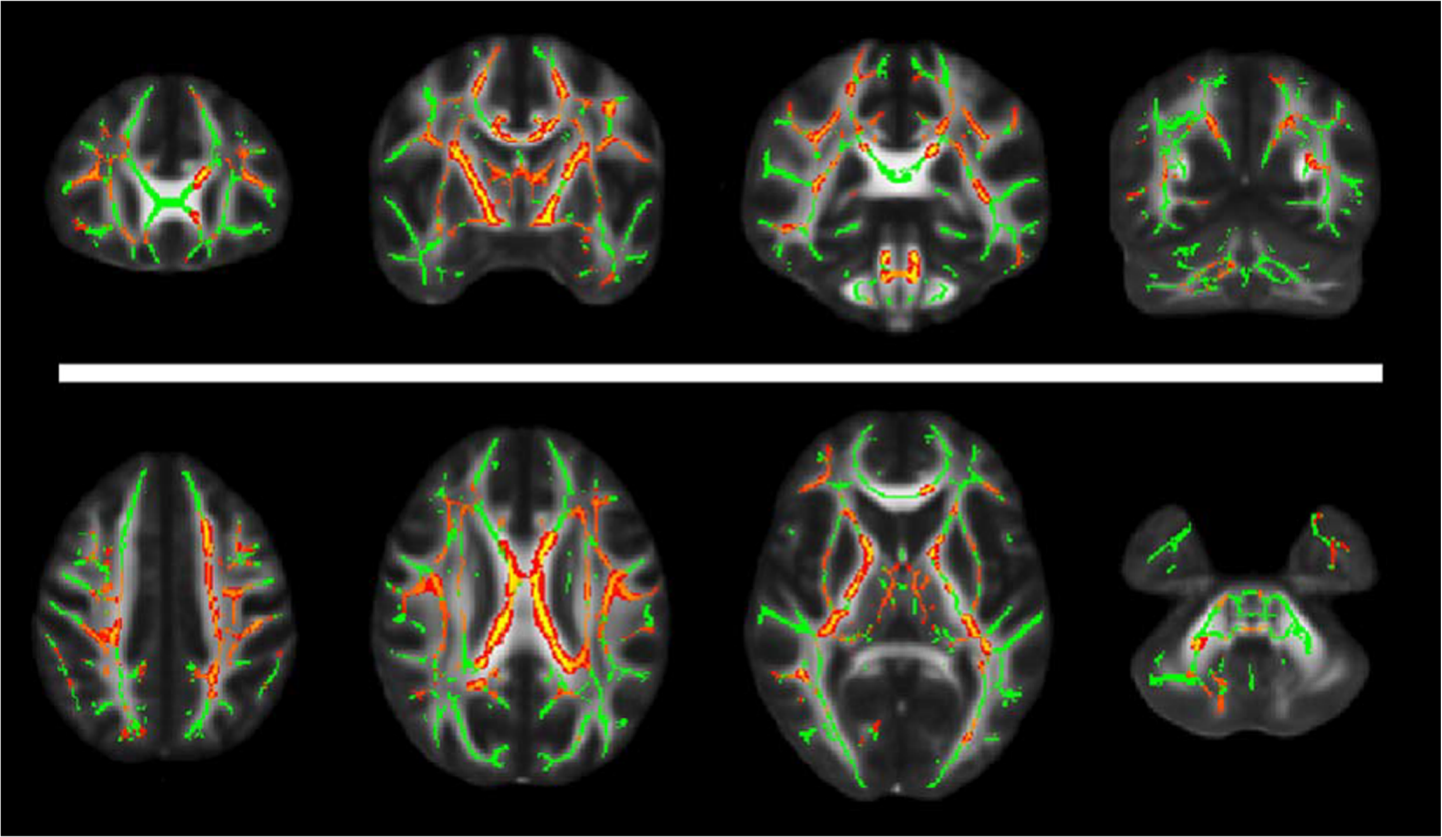
Selected coronal (*upper row*) and axial (*lower row*) slices showing in *red-yellow* the presence of widespread regions of reduced fractional anisotropy in Fabry disease patients compared to healthy controls (results of a tract-based spatial statistics analysis reported in [63])

This extensive pattern of microstructural WM alterations has been also demonstrated using tract-based spatial statistics (TBSS) analyses, which combines the strength of voxel-wise and tractography-based analyses [85]. Indeed, the above-mentioned decreased mean diffusivity values were also coupled to a reduced fractional anisotropy throughout the brain of FD patients [65]. Similarly, a large microstructural WM involvement was recently confirmed by a combined TBSS-functional MRI (fMRI) study, in which extensive areas of reduced fractional anisotropy were found in FD patients, despite the low incidence and load of WMH [63]. These areas included different supratentorial and infratentorial WM regions, with a relative sparing of only few brain regions [63], and were correlated to functional cortical changes, denoting the complexity of physiopathological mechanisms of cerebral involvement in FD [63] (Fig. 4).

These findings are compatible with the hypothesis that vasculopathy could be the leading physiopathological mechanism underlying brain tissue alterations in FD [14], with a microvascular pathology that is likely to cause tissue damage corresponding not only to discrete WM hyperintensities but also to widespread alterations of WM microstructure. However, although different evidence regarding microstructural changes in FD is present in the literature, to date no evidence about possible modification of WM integrity during time is present. For this reason, longitudinal DTI studies are warranted in order to provide information on the natural course of WM microstructural alterations in FD, also offering a valuable tool for monitoring the possible effects of ERT.

### Functional MRI

**Fig. 5:**
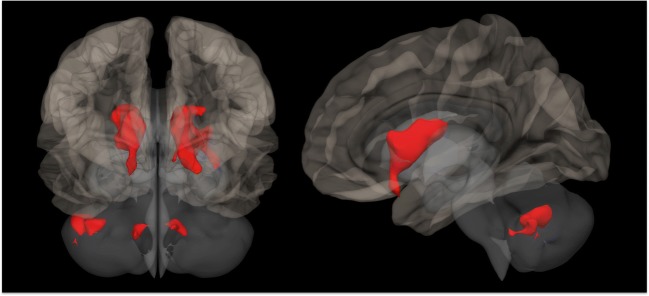
Three-dimensional rendering of a healthy brain in the Montreal Neurological Institute space showing, *in red*, regions of reduced functional connectivity with the motor cortex in Fabry disease patients compared to healthy controls (adapted from [68])

Although widely used in different other pathological conditions, very few fMRI experiments have been performed to date to investigate the presence of possible functional changes in FD patients. The first study was performed by Gavazzi and colleagues [69], by means of a motor task fMRI experiment conducted during repetitive flexion-extension of the last four fingers of the right hand. During the execution of the motor task, FD patients showed an increased activation of additional cortical regions, including the cingulated motor area, the secondary motor area and the primary sensorimotor cortex, with the latter showing a significant correlation with the Fazekas score [69]. This possible involvement of the motor circuits in FD has been further investigated by means of a resting-state fMRI (RS-fMRI), in which the functional connectivity (FC) of both motor cortices have been tested in FD patients without major cerebrovascular events [68]. This study demonstrated the presence of an alteration of the corticostriatal pathway in FD, with a reduction in the FC between motor cortices and the caudate and lenticular nuclei, bilaterally, and between the left motor cortex and cerebellar areas [68] (Fig. 5).

Along with the investigation of motor circuits, RS-fMRI has also been used to study the presence of possible FC alteration of the default mode network (DMN), which is involved in the integration and coordination of sensorimotor and cognitive goal-directed activities [63]. An increased FC between the main hubs of the DMN and different brain areas was proved, either involving the DMN itself or other cerebral and cerebellar cortical regions. These FC modifications also showed a correlation with both the WM microstructural alterations and the patients’ cognitive status [63].

Although only a limited amount of evidence about fMRI alterations in FD is available, diffuse and significant functional changes seem to occur in this condition, thus suggesting a future central role of fMRI in the investigation of cerebral involvement in FD.

### Magnetic resonance spectroscopy, magnetisation transfer ratio and quantitative MRI

Along with the above-mentioned studies, other advanced MRI techniques have also been applied in FD to investigate the physiopathology of CNS involvement in this condition.

Proton MR spectroscopy (^1^H-MRS) offers the possibility to study, detect and quantify in vivo different metabolites in the human brain [86].

One of the first ^1^H-MRS studies in FD patients was performed using a multi-voxel analysis investigating possible changes of the N-acetylaspartate/creatine (NAA/Cr) ratio, indicative not only of neuronal degeneration and loss but also considered as a marker of neuronal dysfunction [70]. This study showed the presence in FD patients of a diffuse reduction of the NAA/Cr ratio in different brain areas, affecting both cortical and subcortical structures [70]. This alteration extended beyond the presence of visible WMH, and was attributed to a possible direct metabolic dysfunction secondary to neuronal Gb3 accumulation rather than a vascular disturbance [70].

Similarly, another multi-voxel analysis demonstrated a reduction of the NAA/Cr ratio in the WM of FD patients, which proved to be also significantly correlated with clinical disability [73].

However, this pattern of reduced NAA/Cr ratio proved to be inconstantly reported in FD patients [71, 87], with a more recent multi-voxel analysis in which no significant metabolite alterations in the normal appearing WM of FD patients were found, suggesting that a detection of CNS involvement using ^1^H-MRS is unlikely in this condition [72].

Beside ^1^H-MRS, other advanced MRI techniques have been sporadically employed in the investigation of the pathological substrate underlying CNS involvement in FD, including magnetisation transfer MRI and quantitative MRI (qMRI) analyses.

Only two studies using magnetisation transfer MRI were performed in FD [73, 74], demonstrating a reduced magnetisation transfer ratio [73] and bound-pool fraction [74] in the normal-appearing WM of FD patients, respectively, suggesting a subtle decrease of myelin density [73, 74].

The use of qMRI techniques allows for a quantitative assessment of brain tissue relaxometry parameters and magnetic susceptibility, measuring physical parameters intrinsically related to tissue microstructure and allowing for an accurate in vivo characterisation of brain tissue pathology [88–90]. Although very promising, a qMRI approach has only been used in two studies in FD, exploring the relevance and the pathological substrate of the pulvinar sign [29] and the possible iron accumulation in the striatonigral pathway of these patients [75]. However, considering the feasibility of this technique for subtle quantitative evaluations, qMRI should be strongly considered in future studies aimed to detect not only markers of neurodegeneration but also the presence of possible glycosphingolipid accumulation in the brain, providing a tool for monitoring disease progression and the treatment’s efficacy.

### Nuclear medicine

Nuclear medicine techniques have been only sporadically applied to assess CNS involvement in FD.

Using a freely diffusible tissue tracer like oxygen-15-labelled water [^15^O-H_2_O], variations in regional cerebral blood flow (rCBF) were assessed in a resting condition, showing an increased rCBF of the posterior and periventricular areas [35] apparently reversible after prolonged ERT, which was then hypothesised to partially normalise this altered cerebrovascular response in FD [77].

This increase in rCBF was later proved to be coupled to a significant reduction of regional cerebral glucose metabolism assessed by 18-fluoro-deoxyglucose (^18^F-FDG) positron emission tomography (PET), more pronounced in regions with a higher risk of developing WMH, in the absence of significant global cerebral glucose metabolism changes [37]. The authors concluded that WMH in FD patients could manifest earlier in hypometabolic but hyperperfused posterior and periventricular regions, suggesting a possible dissociation between metabolism and blood flow as in chronic deep WM metabolic insufficiency [35, 37]. This increase in CBF has been confirmed by a recent arterial spin labelling MRI study, which showed the presence of and increased cerebral flow in FD patients mainly involving the splenium of the corpus callosum, which correlates with the presence of WMH, with a relative sparing of the deep GM [76].

The normal cerebral glucose metabolism distribution in FD patients suggested by Moore and colleagues [78] was confirmed by another study in which areas of reduced metabolism were found only in regions showing infarcts or haemorrhages on MRI scans, with no significant changes during time other than those related to major cerebrovascular events [78].

Considering the functional changes present in FD patients, and given the opportunity to simultaneously investigate metabolic demand and functional activity using the hybrid PET/MR scanners [91], future studies using this technique are warranted. Furthermore, although still under-exploited or limited to anecdotal reports [92], other nuclear medicine techniques such as single photon emission computed tomography (SPECT) could further contribute to improve present knowledge on CNS impairment in FD, with particular reference to recent evidences suggesting a possible link between FD and other neurodegenerative disorders such as Parkinson’s disease [93–95].

## Conclusions

FD is a multiorgan disorder affecting CNS in a variable degree and extent. Conventional neuroradiological findings in this condition are aspecific, and mainly include the presence of WMH and anomalies of the vasculature wall of posterior circulation. Conversely, the once considered pathognomonic and characteristic T1-weighted hyperintensity of the pulvinar should be considered as a very rare neuroradiological finding in FD patients.

The application of different advanced imaging techniques has expanded present knowledge about CNS involvement in this lysosomal storage disorder, shedding new light on the type and mechanisms of cerebral involvement in FD, long considered to be only linked to major cerebrovascular events.

However, different domains still remain unexplored in FD. Therefore, a proper use and combination of different conventional and advanced imaging techniques is warranted, to further help our understanding about CNS physiopathology in this complex systemic condition.

## Abbreviations

CNS: Central nervous system
ERT: Enzyme replacement therapy
GM: Grey matter
FC: Functional connectivity
FD: Fabry disease
Gb3: Globotriaosylceramide
NAA/Cr: N-acetylaspartate/creatine ratio
rCBF: Regional cerebral blood flow
WM: White matter
WMH: White matter hyperintensities
